# Macromolecular Crowding as a Suppressor of Human IAPP Fibril Formation and Cytotoxicity

**DOI:** 10.1371/journal.pone.0069652

**Published:** 2013-07-29

**Authors:** Janine Seeliger, Alexander Werkmüller, Roland Winter

**Affiliations:** Physical Chemistry I - Biophysical Chemistry, Faculty of Chemistry, Technische Universität Dortmund, Dortmund, Germany; University of Maryland School of Medicine, United States of America

## Abstract

The biological cell is known to exhibit a highly crowded milieu, which significantly influences protein aggregation and association processes. As several cell degenerative diseases are related to the self-association and fibrillation of amyloidogenic peptides, understanding of the impact of macromolecular crowding on these processes is of high biomedical importance. It is further of particular relevance as most *in vitro* studies on amyloid aggregation have been performed in diluted solution which does not reflect the complexity of their cellular surrounding. The study presented here focuses on the self-association of the type-2 diabetes mellitus related human islet amyloid polypeptide (hIAPP) in various crowded environments including network-forming macromolecular crowding reagents and protein crowders. It was possible to identify two competing processes: a crowder concentration and type dependent stabilization of globular off-pathway species and a – consequently - retarded or even inhibited hIAPP fibrillation reaction. The cause of these crowding effects was revealed to be mainly excluded volume in the polymeric crowders, whereas non-specific interactions seem to be most dominant in protein crowded environments. Specific hIAPP cytotoxicity assays on pancreatic β-cells reveal non-toxicity for the stabilized globular species, in contrast to the high cytotoxicity imposed by the normal fibrillation pathway. From these findings it can be concluded that cellular crowding is able to effectively stabilize the monomeric conformation of hIAPP, hence enabling the conduction of its normal physiological function and prevent this highly amyloidogenic peptide from cytotoxic aggregation and fibrillation.

## Introduction

**Figure 1:**
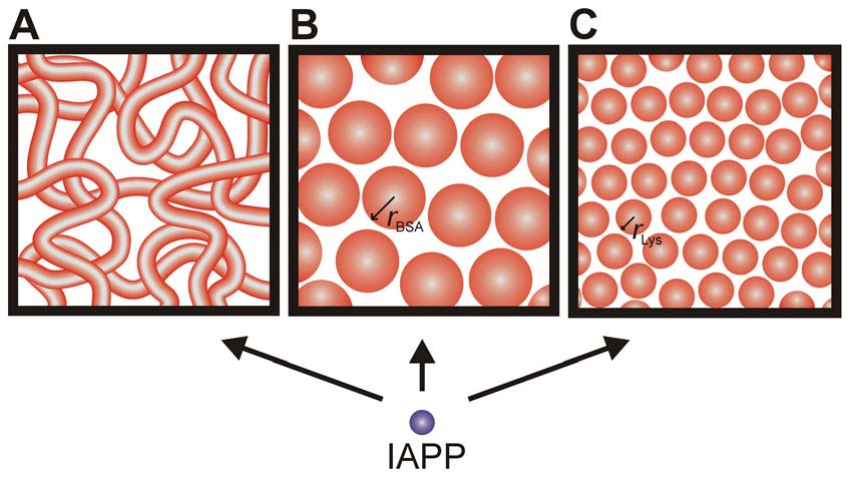
Schematic illustration of the crowder systems' molecular characteristics. **A** High concentrations of Ficoll and dextran exhibit a network-like structure. **B**, **C** The proteins BSA and lysozyme can be described as hard sphere systems with BSA (**B**) having a larger hydrodynamic radius than lysozyme (**C**), leading to differences in the dimensions of void volume.

Over the last decade, phenomena of macromolecular crowding have increasingly gained attention in protein aggregation studies. Crowding studies aim to simulate the high interior concentration of various macromolecules present within the biological cell, whose volume is occupied by proteins and other biopolymers to an extent of about 20–30% [1], [2]. Crowding leads to effects of excluded volume. However, additional factors, such as increased viscosity, reduced diffusion constants and non-specific interactions have been shown to significantly influence the properties of biomolecules in their physiological environment as well [3]–[5]. Typically, two different crowder types are considered: polymers and polysaccharides, such as polyethylene glycol, Ficoll or dextran; and globular proteins, like bovine serum albumin (BSA) or lysozyme. The polymeric crowder Ficoll and dextran are relatively inert, highly soluble, exhibit an average molecular mass of ∼70 kDa and form at high concentrations network-like structures of different viscosities (Figure 1) [6]–[8]. In contrast, BSA and lysozyme can essentially be regarded as hard spheres exhibiting sizes (radii) of *r*_BSA_≈3.1 nm and *r*_Lys_≈1.7 nm, respectively, and are used to monitor additional effects due to non-specific interactions [9]–[11]. Taking both types of agents into account, the trigger of crowding in cellular environments such as cellular network-like fibers and dense protein solutions can be simulated.

Understanding the impact of macromolecular crowding on protein aggregation is of fundamental biomedical importance. Several cell degenerative diseases, such as Alzheimer's disease or type-2 diabetes mellitus, are related to the misfolding, self-association and finally fibrillation of amyloidogenic peptides and proteins, whose natural environment is the crowded cell [12]–[16]. However, most of the *in vitro* studies on amyloid aggregation have been performed in diluted solution which does not represent the complexity of their cellular surrounding and may lead to a different behavior of the amyloidogenic species compared to the *in vivo* situation. Therefore it is appropriate to mimic crowded physiological environments by the addition of macromolecular crowding agents *in vitro* [17]–[25].

In this study, we analyze the influence of macromolecular crowding on the aggregation properties of the highly amyloidogenic human islet amyloid polypeptide (hIAPP), which comprises a length of 37 amino acids. The hIAPP is associated with type-2 diabetes mellitus and is responsible for the disease accompanying β-cell membrane permeabilization and ultimately β-cell loss [14]–[16], [26]–[32]. Utilizing four different polymeric and protein crowders to simulate different components of the cellular environment, we reveal an effective stabilization of non-fibrillar and non-toxic conformations of hIAPP. These effects on the hIAPP fibrillation and the underlying mechanism are studied using fluorescence correlation spectroscopy (FCS), the fluorescence spectroscopic Thioflavin T (ThT) assay, atomic force microscopy (AFM) and attenuated total reflection Fourier-transform infrared (ATR-FTIR) spectroscopy.

## Results

### Diffusion constants as a measure for the constriction of IAPP in crowded solutions

**Figure 2:**
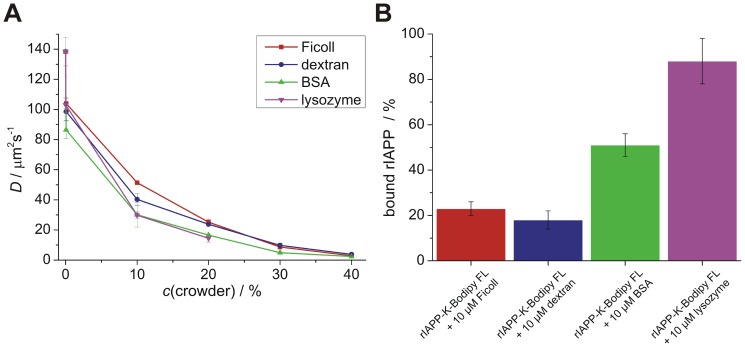
Influence of the crowder type on IAPP restriction and binding. **A** Crowder concentration-dependent lateral diffusion constant, *D*, of 10 µM fluorescent labeled rIAPP (rIAPP-K-Bodipy FL) as monitored by FCS. The polymer-like network forming molecules Ficoll and dextran as well as the proteins BSA and lysozyme were used as crowding reagents at concentrations ranging from 10 µM to 40% (w/v). **B** Content of rIAPP bound to the different crowding agents as analyzed from the FCS results of the equimolar rIAPP-K-Bodipy FL to crowder mixture (10 µM each). Data points are mean values (errors bars indicate standard deviations) of ≥3 individual measurements.

To gain an insight into the way in which the mobility of unstructured monomeric IAPP is constricted within the various crowding solutions and to monitor potential binding of IAPP to the crowding agents, its diffusional properties have been determined. More specifically, the lateral diffusion constant, *D*, of C-terminally Bodipy FL labeled rat IAPP (rIAPP-K-Bodipy FL) has been measured as a function of crowder concentration using fluorescence correlation spectroscopy (FCS) (Figure 2A and Table S1 for details). The rIAPP peptide has been used as it is a non-amyloidogenic homologue of hIAPP, which differs in only six out of 37 amino acids and persists in its monomeric conformation [33], [34], so that changes in the diffusion constants can be solely ascribed to differences in crowding properties rather than to peptide aggregation. Already under non-crowding conditions in a dilute equimolar mixture with any of the crowding reagents the diffusion constant of rIAPP-K-Bodipy FL decreases significantly, indicating non-negligible binding of rIAPP to the crowding reagents. Using a two-component model for the analysis of the FCS data (see Materials and Methods), the amount of rIAPP bound to the individual crowding reagents could be determined (Figure 2B and Table S2 for details). Only a small amount of rIAPP (∼20%) binds to the polymers Ficoll and dextran. In contrast, in solution with BSA nearly 50% of rIAPP and with lysozyme almost all rIAPP is bound to the protein, indicating that non-specific interactions have a substantial impact on IAPP constriction in these protein-crowded environments. By increasing the crowder content, so that strong crowding conditions are reached, a further concentration-dependent, essentially exponential decrease of the diffusion constants is detected for all crowding reagents, with slightly different characteristics, however (Figure 2A). The diffusion constant decreases to significantly smaller values at low crowder concentrations (*c*(crowder) = 10%) for the globular proteins BSA and lysozyme (*D*≈30 µm^2^ s^−1^) compared to the polymer-like crowders Ficoll (*D* = 51 µm^2^ s^−1^) and dextran (*D* = 40 µm^2^ s^−1^). This indicates a markedly stronger constriction in the hard-sphere-like protein solutions, probably owing to the non-specific interactions of these crowders with IAPP and probably also due to the narrower and more homogeneous size distribution of void volumes in dense protein-crowded solutions. However, with increasing crowder concentration, the diffusion constants are approaching similar values for all crowding agents. This general decrease cannot be solely ascribed to intermolecular interactions of crowder and rIAPP as full binding of rIAPP would have been achieved at a 10% crowder concentration already. This additional decrease of the diffusion constants can be attributed to phenomena like excluded volume and increased viscosity.

### Influence of molecular crowding on the hIAPP aggregation and fibrillation reaction

**Figure 3:**
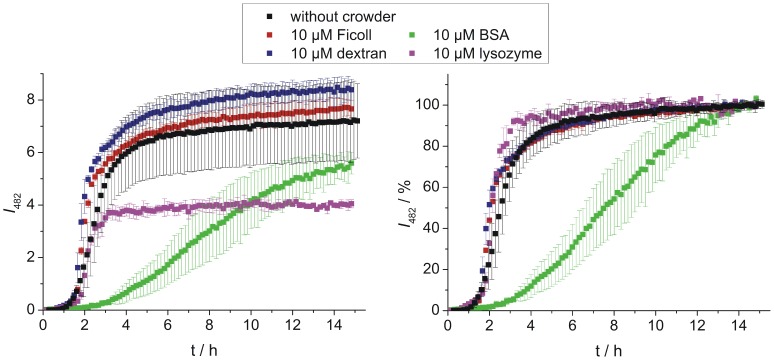
Monitoring crowder binding effects on hIAPP aggregation. Time dependent ThT fluorescence spectroscopic measurements of equimolar solutions of hIAPP and crowding reagents (10 µM each). The left panel shows absolute ThT fluorescence intensities, whereas the corresponding intensity normalized data are presented in the right panel. Data points are mean values (errors bars indicate standard deviations) of ≥6 experiments.

**Figure 4:**
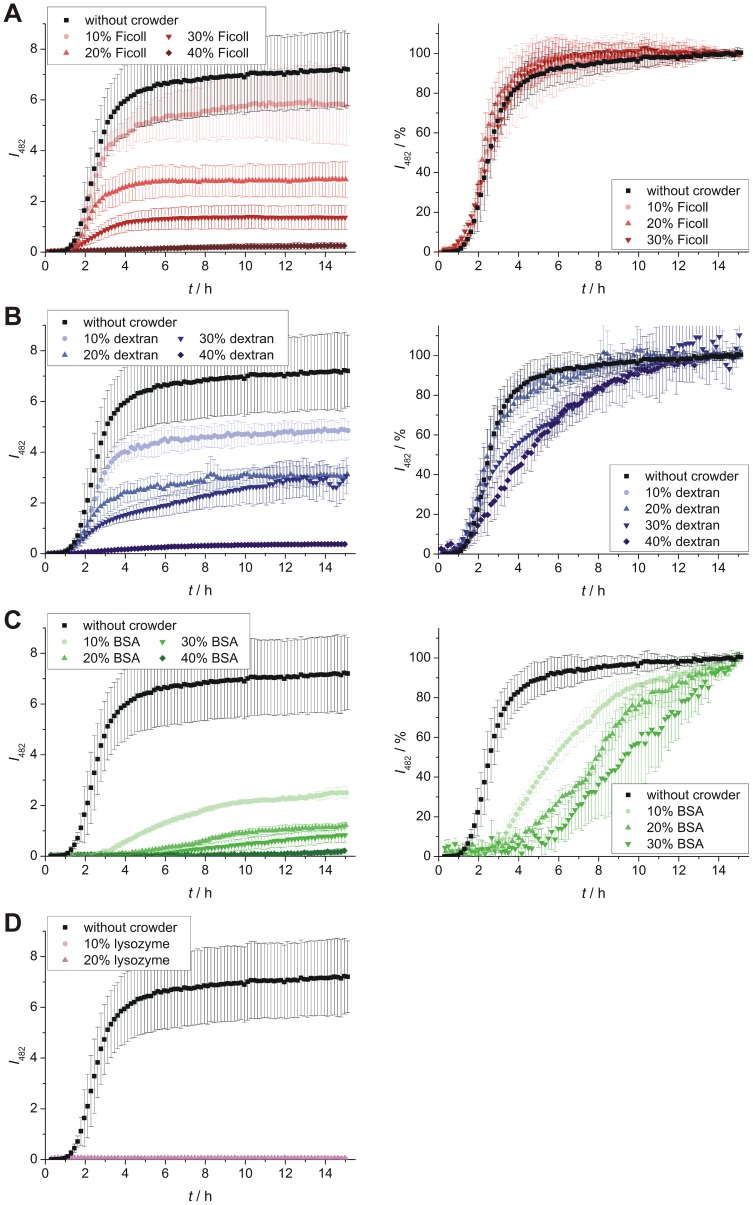
The hIAPP aggregation behavior under highly crowded conditions monitored by ThT fluorescence spectroscopy. Aggregation time courses of hIAPP (10 µM) in solutions using (**A**) Ficoll, (**B**) dextran, (**C**) BSA and (**D**) lysozyme at concentrations of 10–40% (w/v) as crowding reagents. Left panels show absolute ThT fluorescence intensities, whereas the corresponding intensity normalized data are presented in the right panels. Data points are mean values (errors bars indicate standard deviations) of ≥6 experiments.

The influence of macromolecular crowding on hIAPP aggregation has been determined using ThT fluorescence spectroscopy. ThT is a fluorescence dye that exhibits increased fluorescence intensity at 482 nm upon non-covalent binding to amyloid fibrils. For hIAPP and other amyloids, time-dependent ThT fluorescence intensity measurements usually reveal – according to a nucleation and growth type of model – a sigmoidal curve [15], [35], [36]. At first, the interaction of the crowding reagents with hIAPP (10 µM each) was determined under non-crowding conditions to study the effect of crowder-binding on hIAPP aggregation (Figure 3). No significant effect is observed in the case of the polymeric crowders Ficoll and dextran, which is consistent with the finding of rather low binding to IAPP. On the contrary, BSA and lysozyme display already at this low concentration a marked influence on hIAPP aggregation, as the lag time is nearly doubled by BSA (see Table S3 for details) and the maximum fluorescence intensity of ThT is reduced by both proteins. In crowded solutions, a significant crowder-concentration dependent decrease of the ThT fluorescence intensity was detected as a common effect (Figure 4, left panels), indicating that the amount of hIAPP fibrils formed is reduced by all crowding reagents. In the case of very high concentrations of Ficoll, dextran and BSA (40%) as well as for all lysozyme concentrations, the fibrillation reaction of 10 µM hIAPP is even inhibited. Where applicable, data normalized to the final intensity are shown on the right hand side of Figure 4, making it easier to compare the kinetics of the aggregation processes of hIAPP. Though the amount of fibrils decreased, no changes in the kinetics compared to the hIAPP fibrillation reaction in the absence of crowding reagents were found for all concentrations of Ficoll (10–30%) and for low dextran concentrations (10–20%); lag times persist at ∼1.4 h and apparent growth constants, *k*_app_, at ∼2.1 h^−1^. The unchanged kinetics in terms of lag times can be attributed to partial binding of hIAPP monomers to the crowder and/or to a stabilization of hIAPP species in small cavities by confinement. In either case, the remaining population of hIAPP undergoes the usual fibrillation reaction, however, with the result of less fibril production, as it is detected in the ThT measurement. Higher amounts of dextran (30–40%) resulted in an extended elongation phase and a simultaneous loss of the typical sigmoidal shape of the ThT curve. This indicates a more complex aggregation kinetics, which probably involves next to effects like binding and excluded volume also diffusion-limited steps as the viscosity of dextran is significantly higher at these concentrations compared to the viscosity of the Ficoll solution. In the case of BSA as crowding reagent, prolonged lag phases – up to 5.1 h – as well as decreased *k*_app_ values – as low as 0.5 h^−1^ – have been determined. Effects like excluded volume, high viscosity and drastically reduced diffusivities together with significant binding of hIAPP monomers to BSA seem to slow down nuclei formation as well as fibril elongation of hIAPP.

Taken together, these results still reveal the existence of hIAPP fibrillation in the crowded milieu, but the amount of accumulated fibrils is dramatically reduced in a crowder-concentration and crowder-type dependent manner. This suggests that stable hIAPP species, which may be bound to the crowder or stabilized in the confinement, are formed which are not able to fibrillate.

### Characterization of the accumulated hIAPP species

**Figure 5:**
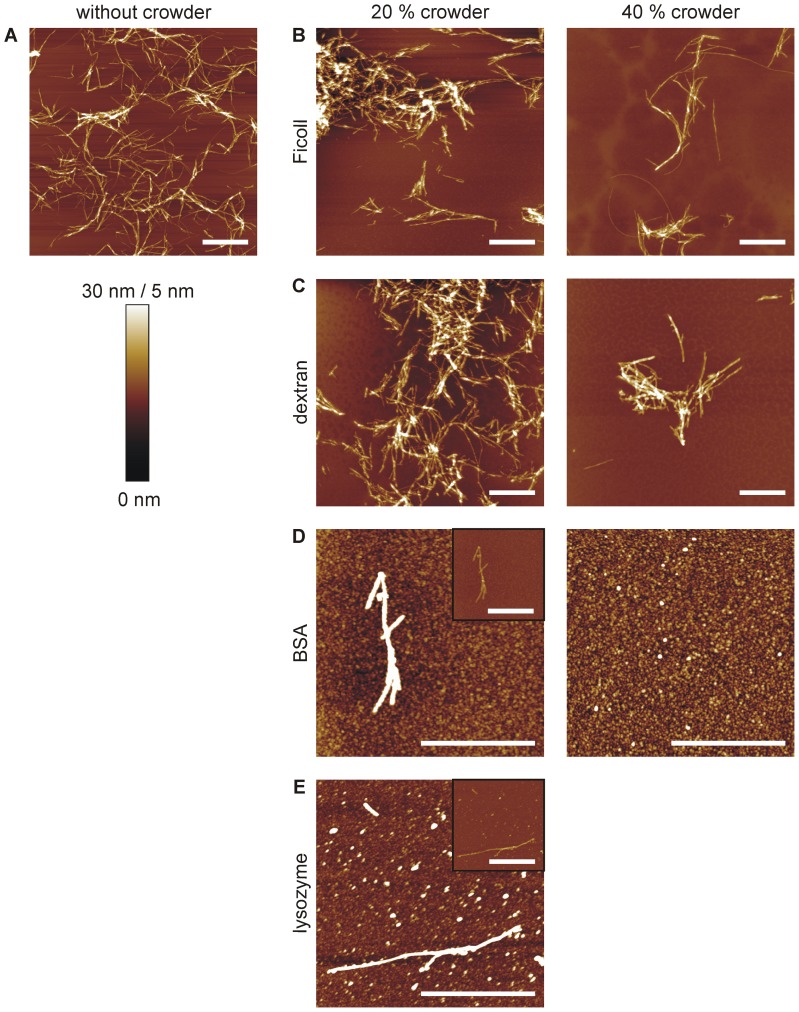
Structural differences of hIAPP aggregates upon incubation in crowded environments as detected by AFM. AFM images showing the structures of 50 µM hIAPP after ∼15 h of incubation in solutions with and without crowding reagents. **A** hIAPP only. **B**–**E** Incubation of hIAPP in 20% or 40% (w/v) of Ficoll, dextran, BSA and lysozyme. For an easier comparison of the hIAPP fibril sizes, insets in the left images of **D** and **E** show the same magnification as the other images displaying fibrils. All scale bars correspond to 1 µm. The color scale represents a height of 30 nm for images **A**–**C** and 5 nm for images **D** and **E**.

To identify which kind of hIAPP species – whether oligomers, protofibrils or mature fibrils – are populated in the highly crowded solutions, AFM measurements were conducted (Figure 5). As a higher hIAPP concentration was needed for these measurements, samples of 50 µM hIAPP after 15 h of incubation in crowded solutions were used. The corresponding time-lapse ThT data can be found in the Figure S1, showing similar results as detected for the lower 10 µM hIAPP concentration with minor changes in the kinetics and strength of the inhibitory effect of the crowder, only. The AFM images reveal the formation of mature fibrils displaying heights of around 6±2 nm (see Table S4 for details) in the case of hIAPP incubation in the absence of crowding reagent and for 20 and 40% Ficoll and dextran (Figure 5A–C). Although AFM is not suitable itself in precisely quantifying the amount of fibrils formed, a qualitative decrease in the numbers of fibrils present in samples of 40% Ficoll and dextran has clearly been observed, which is consistent with the ThT results of 50 µM hIAPP. Conversely, mainly globular species were obtained for hIAPP incubated in BSA and lysozyme solutions (Figure 5D, E). These hIAPP species formed could be divided into two fractions: smaller oligomers (*h*≈0.8±0.5 nm) were detected in all images containing BSA or lysozyme as crowding reagent, whereas additional larger particles displaying heights of nearly fibrillar size were solely seen in 40% BSA (*h* = 4.4±0.6 nm) and 20% lysozyme (*h* = 4.9±1.3 nm) solutions. These smaller globular species could present hIAPP oligomers, which have been shown before to exhibit mean heights of ∼0.8 nm [31]. However, as a strong interaction with the protein-crowder has been detected by FCS and ThT measurements, the smaller as well as the larger globular species could also display co-aggregates of hIAPP with BSA or lysozyme. Nevertheless, individual mature hIAPP fibrils were still found in 20% BSA and 20% lysozyme solutions revealing heights of 5.2±1.0 nm and 6.1±2.0 nm, respectively. Altogether, the AFM data clearly demonstrate that less fibrils are formed in crowded environments, which however all, even the very few fibrils formed in protein-crowded solution, exhibit a similar morphology (height, length) compared to fibrils formed from diluted solution. Furthermore, instead of fibrils more globular species appear in the protein-crowded solutions.

### The mechanism of mature fibril formation is not affected by crowding

**Figure 6:**
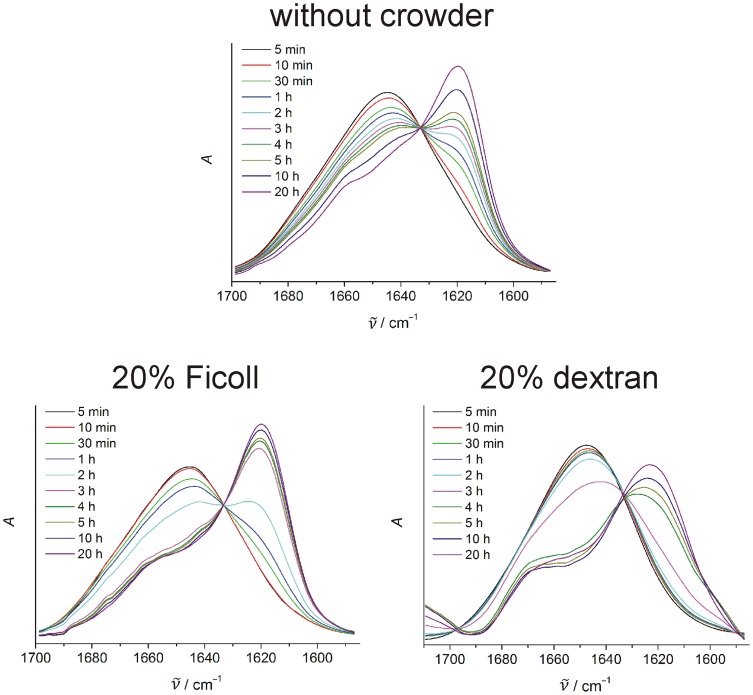
The hIAPP aggregation mechanism remains unaffected by crowding. Area-normalized ATR-FTIR spectra showing time-dependent shifts of the amide-I′ band of 10 µM hIAPP, 10 µM hIAPP in 20% Ficoll solution and 10 µM hIAPP in 20% dextran solution.

Additional information on the mechanism of the hIAPP fibril formation has been gained by ATR-FTIR spectroscopy, which allows detection of secondary structural changes of the peptide from the analysis of its amide-I′ band. Area-normalized spectra of the amide-I′ band of pure hIAPP as well as of hIAPP in crowded solutions of 20% Ficoll and dextran are depicted in Figure 6. The crowding reagents BSA and lysozyme cannot be studied using this technique as both are proteins themselves, whose amide-I′ bands would overlap with the one of hIAPP. For all measured samples, the hIAPP amide-I′ band maximum shifts from ∼1645 cm^−1^ to ∼1620 cm^−1^ with time, with an isobestic-like point appearing at ∼1633 cm^−1^. This points towards a secondary structural change from an initially disordered and partially α-helical conformation to a structure mainly consisting of intermolecular β-sheets, thus indicating that fibrillation of hIAPP occurs via a similar mechanism in all samples. As can be seen from the primary spectra (cf. Figure S3), changes in band intensities are observed, only. The absorbance intensity of the spectra decreases markedly in the crowded environment, indicating a marked decrease of the amount of hIAPP forming fibrils, which is consistent with the AFM and ThT spectroscopic data.

### How crowding affects hIAPP cytotoxicity

**Figure 7:**
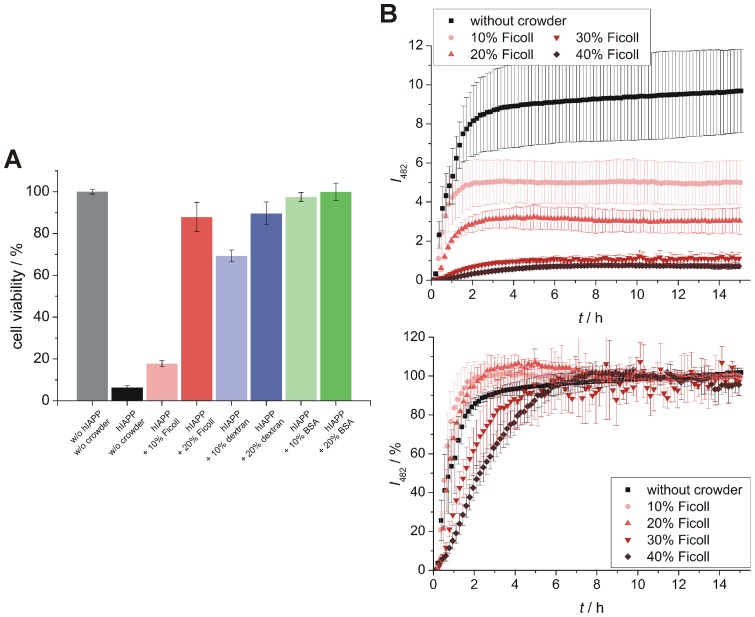
Effect of crowding on hIAPP cytotoxicity and on cell membrane enhanced hIAPP aggregation. **A** Cell viability of pancreatic β-cells after exposure to hIAPP (10 µM) in crowded environments of different type (Ficoll, dextran, BSA) and concentration (10 and 20% (w/v)). As controls, viability of β-cells incubated without hIAPP and crowder reagents (grey bar) as well as with hIAPP (10 µM) without crowding reagents (black bar) are shown. **B** Time dependent ThT fluorescence spectroscopy assay of 10 µM hIAPP in a solution containing 0.5 mg/mL cell membrane vesicles (lipids extracted from the pancreatic β-cell line INS-1E) and 10 to 40% Ficoll as crowding reagent. The upper panel shows absolute ThT fluorescence intensities; whereas the corresponding normalized data are presented in the lower panel. Both data sets show mean values (errors bars indicate standard deviations) of ≥6 experiments.

The main issue regarding hIAPP aggregation and fibril formation is its association with type-2 diabetes mellitus and the related β-cell membrane permeabilization. To reveal the still unknown effect of crowding solutions on hIAPP cytotoxicity, a WST-1 cell viability assay was applied to the pancreatic β-cell line INS-1E (Figure 7A). As control, β-cells incubated without hIAPP and crowding reagents (grey bar) showed 100% viability, whereas only 6% of the cells remained alive after incubation in the presence of hIAPP and absence of any crowding reagent (black bar). If Ficoll (red bars) or dextran (blue bars) were added to the hIAPP solutions, the cell viability was increased in a concentration-dependent manner, with dextran showing a stronger positive effect than Ficoll. Remarkably, cells incubated with hIAPP and BSA as crowding reagent (green bars) showed full cell viability. These cell viability data perfectly correlate with the amount of accumulated fibrils as detected by the corresponding ThT and AFM measurements (in the presence and absence of lipid vesicles). The more fibrils form, the lower is the β-cell viability. Notably, the stabilized globular hIAPP species do not exhibit any measurable toxicity.

To reveal the impact of cellular membranes on hIAPP aggregation in the absence and presence of crowding agents, additional ThT measurements of solutions containing vesicles of extracted β-cell membrane lipids (from INS-1E cells) and Ficoll as representative crowding reagent were also carried out (Figure 7B). The main influence observed in the presence of vesicles composed of the natural lipids was a significant reduction of the lag phase (cf. Figure 4A). However, this well-known effect of accelerated hIAPP fibrillation in lipid environments [16], [28], [29] affected the non-crowded and the crowded hIAPP solutions in the same way.

## Discussion

In this study two polymeric network-forming and two globular protein crowding reagents have been used to explore the effects of crowding on the aggregation properties of hIAPP. A dramatically crowder-concentration and type-dependent reduction, and at very high crowder concentrations, even full inhibition of fibril formation is observed. The still formed hIAPP fibrils retain their usual morphology, however. Furthermore, at least in the protein-crowded environments, a second kind of hIAPP species has been detected by AFM displaying globular hIAPP monomers or oligomers, which may be bound to the crowder or stabilized in the crowder-confinement and hence is no longer able to fibrillate. Cytotoxicity measurements on the pancreatic β-cell line INS-1E have revealed that the reduction of hIAPP fibrils leads to reduced cytotoxicity, indicating that the globular hIAPP species initiated by crowding are non-toxic.

It has recently been discussed that monomeric hIAPP, which is usually thought to be natively unfolded, can also adopt transiently different structural motifs such as an α-helical segment or an antiparallel β-hairpin [37]–[39]. As it is predicted that crowding reagents may stabilize compact conformations due to the excluded volume effect [40]–[42], such partially folded – and potentially non-amyloidogenic – hIAPP conformations might be stabilized by the crowding agents, thereby leading to the observed stable non-toxic species. In fact, the stabilization of such partially folded conformations could be responsible for the prolonged lag phases observed in the ThT assay as well. Similar results have been obtained for insulin and bovine core histones at neutral pH [19] – these proteins are larger and considerably less aggregation-prone than hIAPP, however. In contrast, most of the other highly amyloidogenic peptides, like α-synuclein, display accelerated aggregation upon addition of crowding reagents [18].

**Figure 8:**
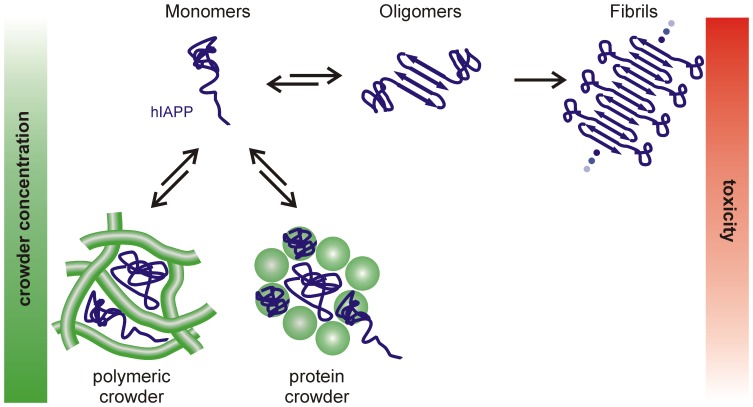
Competing reaction pathways of hIAPP aggregation under crowding conditions. The horizontal path displays the usual hIAPP aggregation mechanism from a natural monomeric disordered structure via formation of nuclei and oligomers to fibril accumulation. Vertically, the competing crowder type dependent stabilization of globular non-toxic off-pathway species is depicted.

Taking all the data together, two competing processes have been uncovered in the crowded solutions, which are schematically visualized in Figure 8. The horizontal reaction path displays the usual hIAPP aggregation mechanism from a natural monomeric structure via formation of nuclei and oligomers to fibril accumulation. This hIAPP aggregation pathway is competing with the stabilization of globular non-toxic off-pathway species of hIAPP by crowding in a concentration and crowder-type dependent manner (Figure 8, vertical reaction profile). Owing to the fact that IAPP is at least to some extent binding to all utilized crowding reagents, the effects of particular contributions to macromolecular crowding, such as exclusively the excluded volume, cannot easily be extracted. Rather, multiple contributions like non-specific interactions as well as excluded volume, increased viscosities and reduced diffusion constants may have to be taken into account. However, due to the fact that IAPP binds only in low amounts to Ficoll and dextran, and in high amounts to BSA and lysozyme, the effects of excluded volume and non-specific interactions can be roughly discriminated. In crowded solutions of polymeric macromolecules, effects like excluded volume, increased viscosity and reduced diffusion constants seem to be the major components for the stabilization of the hIAPP species, whereas non-specific interactions seem to be predominant in protein-crowded environments. In line with recent studies [4], [21], [23], [43]–[45], this argues for a balanced effect of excluded volume and non-specific interactions in crowded environments where the net effect depends on the relative strength of either contribution.

The slightly different effect of Ficoll compared to dextran can be assigned to the difference in viscosity of both solutions at high concentrations, where dextran shows a significantly higher viscosity than Ficoll owing to a different network building block. Dextran is a highly branched polysaccharide made of glucose molecules, whereas Ficoll is a sucrose-polymer formed by copolymerization of sucrose with epichlorohydrine. The variations in hIAPP-aggregation properties in the crowded environment of BSA compared to lysozyme, i.e., the elongation of the lag phase of hIAPP and the deceleration of fibril elongation by BSA or the significantly stronger suppression of hIAPP aggregation in lysozyme solutions, originate from different phenomena. The differences in hIAPP binding as revealed by the FCS measurements could lead to the stronger inhibition of aggregation by lysozyme. Moreover, the different sizes of these two proteins (radii *r*_BSA_≈3.1 nm, *r*_Lys_≈1.7 nm) [10] may also be important as significantly smaller cavities of dense lysozyme solutions may almost completely disable aggregation and fibrillation of hIAPP. The elongated lag phase and decelerated fibril elongation of hIAPP by BSA could be explained by its recently observed chaperone-like activity in the prevention of aggregation and amyloid fibril formation of transthyretin [46]. Hence, BSA may be able to partially bind, stabilize and convert hIAPP back to its monomeric conformation.

## Conclusions

These studies reveal a marked influence of crowding on the aggregation mechanism and the kinetics of the aggregation path of hIAPP, and hence also on the toxicity of the peptide. The cytotoxicity data demonstrate non-toxic effects for stabilized off-pathway species, but reveal high toxicity for the path through the conventional fibrillation mechanism (Figure 8). Furthermore, the data indicate that cellular crowding is not only an effect of excluded volume but also strongly involves non-specific interactions. As the natural environment of hIAPP is the crowded milieu of the cell, our study suggests that cellular crowding is able to stabilize the monomeric unstructured conformation of hIAPP, hence enabling the conduction of its normal physiological function and preventing it from cytotoxic aggregation and fibrillation. Moreover, these data further our understanding why amyloid formation essentially occurs extracellular, where the crowding is less pronounced, in agreement with theoretical predictions [47], [48].

## Materials and Methods

### Crowder specification and peptide preparation

The crowding reagents Ficoll® PM 70, dextran from *Leuconostoc* spp. M_r_ ∼70,000, albumin from bovine serum (BSA) and lysozyme from chicken egg white were purchased from Sigma-Aldrich. Human amylin (hIAPP) was obtained from Merck Millipore. Fluorescence labelled rat IAPP (rIAPP-K-Bodipy FL) was acquired from Peptide Specialty Laboratories. To disaggregate any preformed IAPP oligomers or fibrils, the peptides were first dissolved in 1,1,1,3,3,3-hexafluoro-2-propanol (HFIP) yielding a concentration of 0.5 mg/mL, and incubated for at least 1 h. The required amount of peptide was dried by lyophilisation, dissolved in the appropriate buffer and immediately used for the measurements.

### Preparation of cell membrane lipid vesicles

The INS-1E cells (a gift from the group of Dr. Pierre Maechler, Geneva University Hospital, Switzerland [49]) were cultured and their cell membrane's lipids were isolated, quantified and analyzed as previously described [16]. Lipid vesicles were prepared by dissolving the appropriate amount of dried isolated cell membrane lipids to a concentration of 0.5 mg/mL in ThT-buffer (10 mM NaH_2_PO_4_, 10 µM ThT, pH 7.4) containing 10 to 40% (w/v) of the corresponding crowding reagent. After sonication for 15 min, five freeze-thaw cycles were performed (thawing at 70°C), followed by additional 5 min of sonication. The vesicle solution obtained was cooled to room temperature and directly used for the ThT fluorescence spectroscopy measurements.

### Thioflavin T (ThT) fluorescence spectroscopy

The hIAPP (10 µM or 50 µM) was incubated in ThT-buffer (10 mM NaH_2_PO_4_, 10 µM ThT, pH 7.4) containing 10 µM or 10 to 40% (w/v) of the corresponding crowder in a 96-well plate at a stable temperature of 25°C. For studying the effect of lipid vesicles on hIAPP aggregation in crowded environments, ThT-buffer containing cell membrane lipid vesicles and the appropriate crowder concentration (prepared as described above) was used. The ThT fluorescence intensity was measured every 10 min after shaking for 10 s at an emission wavelength of 482 nm after excitation at 440 nm using a plate reader (Infinite M200, Tecan). All data were background corrected by subtraction of the ThT fluorescence intensity of the corresponding buffer without hIAPP. For normalization, the intensity at every point was divided by the plateau intensity, which was defined as the average intensity in the last hour of the measurement. Data analysis was carried out as described previously [50] using the software Origin (OriginLab Corporation).

### Atomic force microscopy (AFM)

After running a ThT fluorescence spectroscopy assay of hIAPP (50 µM) for 15 hours under different crowding conditions, aliquots were taken from the 96 well plate, diluted to 25 µM with water and deposited on freshly cleaved mica. The samples were dried with a stream of nitrogen, rinsed with water, again dried with a stream of nitrogen and freeze-dried overnight. Controls of crowder solutions in the absence of hIAPP on mica were prepared in the same way (Figure S2).

AFM measurements were performed in the tapping mode on a MultiMode scanning probe microscope equipped with a NanoScope IIIa controller (Digital Instruments) using an E-Scanner (scan size 15 µm×15 µm) and a MMMC cantilever holder (both Veeco Instruments) equipped with a silicon SPM sensor (PPP-NCHR, NanoAndMore). The dried samples were scanned in air with drive frequencies around 240 kHz and drive amplitudes between 15 and 379 mV. All AFM measurements were performed at room temperature using scan rates between 1.0 and 1.5 Hz. Height and amplitude images of sample regions were acquired with resolutions of 512×512 pixels. For image analysis and processing, the software Nanoscope 5 (Veeco Instruments) was used.

### Attenuated total reflection Fourier-transform infrared (ATR-FTIR) spectroscopy

ATR-FTIR spectra were recorded using a Nicolet 6700 infrared spectrometer equipped with a liquid nitrogen cooled MCT (HgCdTe) detector and an ATR out-of compartment accessory consisting of a liquid jacketed ATR flow-through cell (Thermo Scientific) with a trapezoidal Si-crystal (80×10×4 mm^3^, angle of incidence: 45°, Resultec). For each FTIR spectrum 128 scans with a spectral resolution of 2 cm^−1^ were taken. The ATR flow-cell was tempered to 25°C before a buffer spectrum was collected, and a solution of 10 µM hIAPP in 10 mM NaH_2_PO_4_, pH 7.4 in D_2_O containing 20% (w/v) of the corresponding crowding reagent was injected. Spectra were taken every 5 min for a period of 20 hours. Data analysis was carried out using the GRAMS software (Thermo Electron). After buffer subtraction, the spectra were baseline corrected between 1710 and 1585 cm^−1^ and, if required, normalized to the amide-I′ band area.

### Cell viability assay

INS-1E cells were seeded into a 96 well plate at 10^5^ cells/mL and grown for 72 h before exposure to the crowded hIAPP solutions. These solutions consisted of 10 µM hIAPP together with 10–20% crowding reagent in cell culture medium [RPMI 1640 (with 2 mM glutamine) supplemented with 5% FCS, 10 mM HEPES, 1 mM sodium pyruvate, 50 µM 2-mercaptoethanol, 100 U/mL penicillin and 100 µg/mL streptomycin, pH 7.4]. After 24 h of incubation at 37°C under 5% CO_2_ the solutions were removed, cells were washed once with fresh medium and incubated for another 24 h with a WST-1 solution (2,5% Cell Proliferation Reagent WST-1(Roche), 7,5% PBS, 90% culture medium). The absorbance was read at 450 nm (reference at 630 nm). Percentage cell viability was calculated based on the absorbance measured relative to that of cells exposed to culture media containing the crowding reagents without hIAPP. Compared to cells grown in culture medium alone, cells exposed to media containing crowding reagents displayed full viability (Figure S4).

### Fluorescence correlation spectroscopy (FCS)

FCS experiments were carried out on an extended confocal microscope system (MRC-1024, Biorad with the inverted microscope Eclipse TE-300 DV, Nikon), which enables fluorescence excitation in a non-scanning mode. The 488 nm line of a krypton-argon ion laser was used for excitation of Bodipy FL labelled rIAPP and focussed by a Nikon apochromat 1.2 NA 60× water-immersion objective. The laser-power was set to 35 µW. Fluorescence emission was collected through the same objective and transmitted to an optical module at the bottom exit of the microscope, directed through an emission filter (520/35, AHF) and finally focussed onto the entrance aperture of a multimode fibre (10 µm diameter). Online autocorrelation functions *G*(*τ*) were calculated from the intensity time traces by an ALV-7002 multiple tau digital correlator (ALV-Laser GmbH).

The autocorrelation curves obtained were fitted to a three-dimensional diffusion model assuming a Gaussian detection volume:
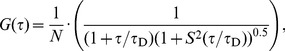
(1)where *N* corresponds to the average number of fluorescent particles within the detection volume and *S* is a parameter describing the ratio between the lateral and axial radii,*ω*_0_ and *z*_0_, of the confocal volume (*S* = *ω*_0_/*z*_0_ = 0.2).*τ*_D_ is the diffusion time, which is related to the lateral diffusion coefficient *D* through the expression
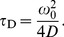
(2)An example is shown in Figure S5. For investigation of the non-specific interactions between IAPP and the different crowder molecules we also performed binding studies under non-crowding conditions. Data obtained here were fitted to a two-component 3D-diffusion model:
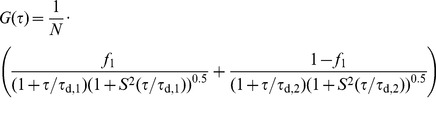
(3)The symbols *τ*_d,1_ and *τ*_d,2_ refer to the diffusion times of free and crowder-bound rIAPP-K-Bodipy FL peptide, respectively, whereas *f*_1_ describes the fraction of free rIAPP particles connected to diffusion time *τ*_d,1_. Both diffusion times were kept constant during the fitting procedure, yielding the fraction of free diffusing rIAPP. The diffusion time *τ*_d,1_ was gained from the measurement of pure rIAPP-K-Bodipy FL peptide. Diffusion times *τ*_d,2_ of crowder-bound rIAPP were assumed to be equal to the diffusion times of the crowding component alone and were calculated from literature values of *D*, e.g. in case of dextran (23 µm^2^ s^−1^) [51], BSA (61 µm^2^ s^−1^) [52] and lysozyme (105 µm^2^ s^−1^) [53]. The diffusion constant of Ficoll (44 µm^2^ s^−1^) was calculated from the Stokes-equation using a hydrodynamic radius of 5.5 nm [54], [55].

The system was calibrated by measuring the autocorrelation curve of a rhodamine dye (Invitrogen, *D* = 280 µm^2^ s^−1^ [56]), yielding a lateral radius *ω*_0_ of 198±2 nm. The rIAPP-K-Bodipy FL peptide was dissolved in buffer (10 mM NaH_2_PO_4_, pH 7.4) containing the respective crowding reagent (Ficoll, dextran, BSA, and lysozyme, 10 µM or 10–40% (w/v)) at a concentration of 10 µM. Total volumes of 50 µL were spread on a glass-cover slip and autocorrelation curves were recorded 20 µm above the glass surface for 100 s. All measurements were performed at room temperature.

## Supporting Information

Figure S1 **Aggregation time course of hIAPP in crowded solutions as applied to AFM measurements.** hIAPP (50 µM) was incubated and monitored under ThT-assay conditions in different crowding solutions as shown here, before aliquots of this reaction were taken after ∼15 h for AFM visualisation of the species obtained. The left panel shows absolute ThT fluorescence intensities, the corresponding normalized data are presented in the right panel.
(TIF)

Figure S2 **AFM images of mica surfaces incubated with crowder solutions in the absence of hIAPP.** Representative images of 40% dextran (left) and 40% BSA (right) incubated under the same conditions as in the presence of hIAPP are displayed. The images are shown in the same sizes and magnifications as the corresponding images with hIAPP in the main manuscript. In both cases as well as for pure Ficoll and pure lysozyme only a thin layer of crowding material was detected on the flat mica surface, which is significantly different from the detected hIAPP fibrils and globular species. Scale bars correspond to 1 µm.
(TIF)

Figure S3 **Representative primary ATR-FTIR spectra.** Spectra of 10 µM hIAPP, 10 µM hIAPP in 20 % Ficoll solution and 10 µM hIAPP in 20 % dextran solution after buffer subtraction and baseline correction.
(TIF)

Figure S4 **Cell viability of pancreatic β-cells (INS-1E cell line) after exposure to crowding reagents without hIAPP.** Crowder of different type (Ficoll, dextran, BSA, lysozyme) and concentration (10 and 20% (w/v)) were used and as a control, viability of β-cells incubated without hIAPP and any crowder (grey bar) is shown.
(TIF)

Figure S5 **Normalized autocorrelation curves of rIAPP-K-Bodipy FL in Ficoll solutions and the corresponding fits using a free 3D diffusion model.**
(TIF)

Table S1 **Crowder concentration-dependent lateral diffusion constants,** ***D*** **, of 10 µM fluorescent labeled rIAPP (rIAPP-K-Bodipy FL) as monitored by FCS.**
(DOCX)

Table S2 **Fraction of crowder-bound rIAPP-K-Bodipy FL as derived from the two-component 3D-diffusion analysis.**
(DOCX)

Table S3 **Crowding molecule and crowder concentration dependent lag times and growth rate constants for the aggregation/fibrillation process of 10 µM hIAPP.**
(DOCX)

Table S4 **Heights of hIAPP oligomers and fibrils as detected by AFM.**
(DOCX)

Supporting Information S1 **Additional information to the ThT assay results of the samples used for atomic force microscopy (AFM) measurements.**
(DOCX)
