# Supplementary figures and images for: Macromolecular Crowding as a Suppressor of Human IAPP Fibril Formation and Cytotoxicity
Source: PLoS One. 2013 Jul 29;8(7):e69652. doi: 10.1371/journal.pone.0069652 (PMC3726762; doi:10.1371/journal.pone.0069652)

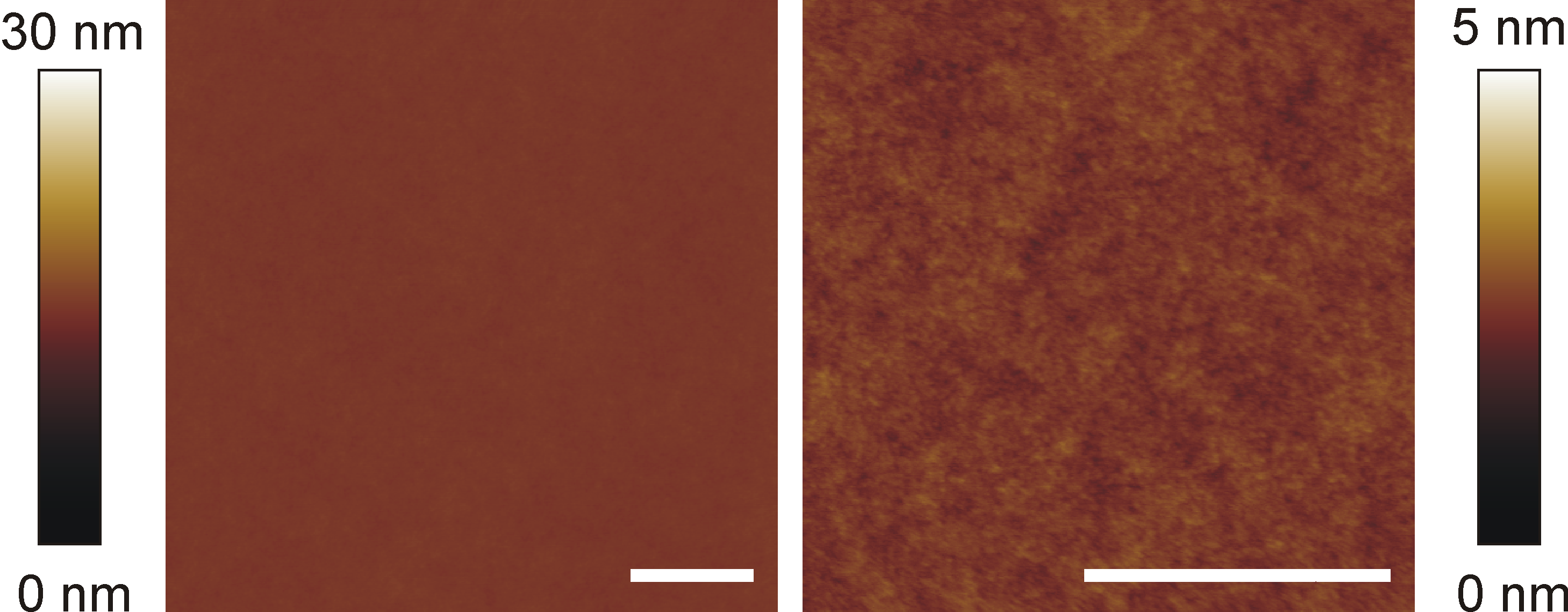

Supplement: Figure S2 — AFM images of mica surfaces incubated with crowder solutions in the absence of hIAPP. Representative images of 40% dextran (left) and 40% BSA (right) incubated under the same conditions as in the presence of hIAPP are displayed. The images are shown in the same sizes and magnifications as the corresponding images with hIAPP in the main manuscript. In both cases as well as for pure Ficoll and pure lysozyme only a thin layer of crowding material was detected on the flat mica surface, which is significantly different from the detected hIAPP fibrils and globular species. Scale bars correspond to 1 µm. (TIF) [file pone.0069652.s002.tif]

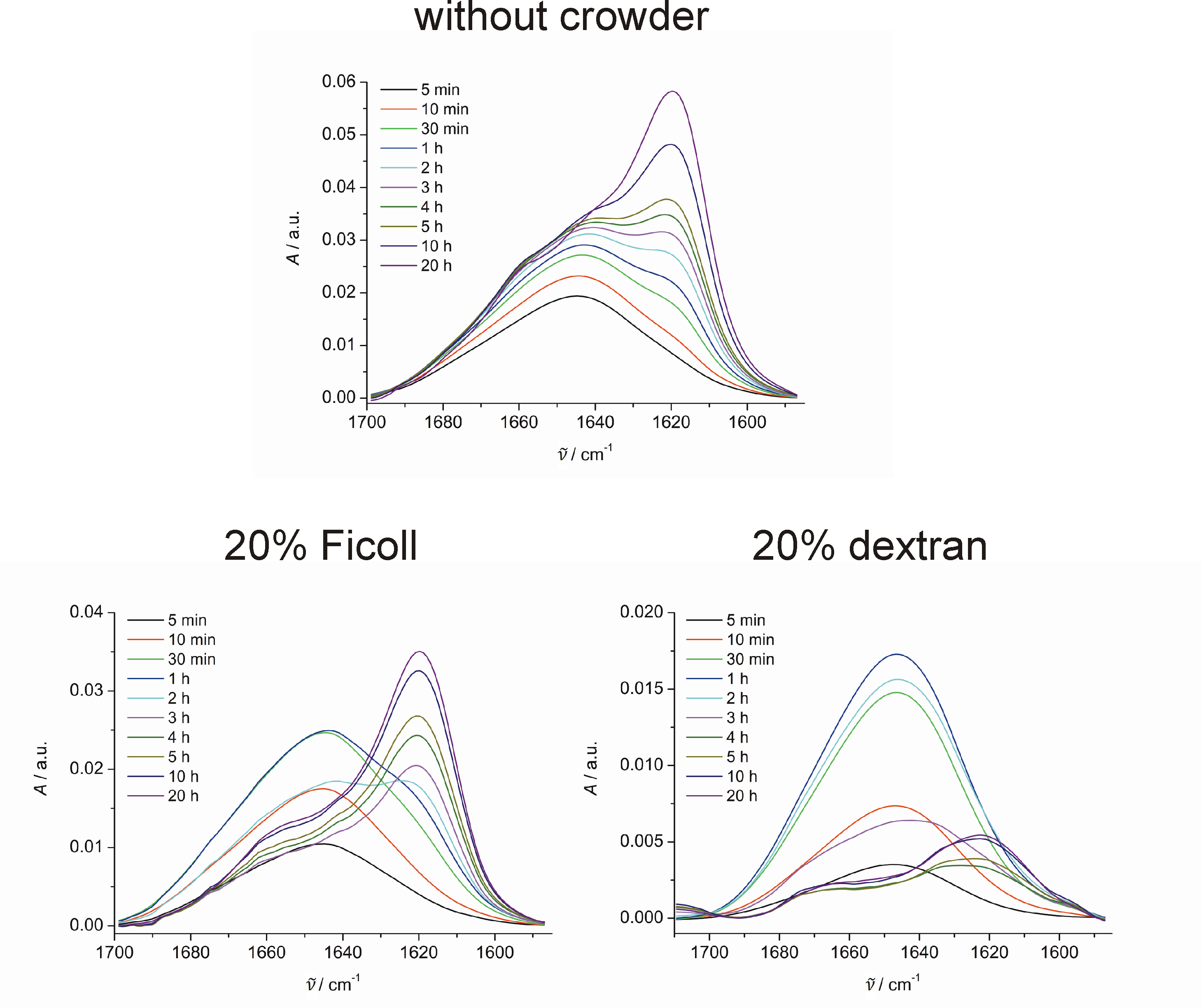

Supplement: Figure S3 — Representative primary ATR-FTIR spectra. Spectra of 10 µM hIAPP, 10 µM hIAPP in 20 % Ficoll solution and 10 µM hIAPP in 20 % dextran solution after buffer subtraction and baseline correction. (TIF) [file pone.0069652.s003.tif]
