# Supplementary material for: Macromolecular Crowding as a Suppressor of Human IAPP Fibril Formation and Cytotoxicity
Source: PLoS One. 2013 Jul 29;8(7):e69652. doi: 10.1371/journal.pone.0069652 (PMC3726762; doi:10.1371/journal.pone.0069652)
Supplement: Table S1 — Crowder concentration-dependent lateral diffusion constants, D , of 10 µM fluorescent labeled rIAPP (rIAPP-K-Bodipy FL) as monitored by FCS. (DOCX) [file pone.0069652.s006.docx]

**Table S1.** Crowder concentration-dependent lateral diffusion constants, *D*, of 10 µM fluorescent labeled rIAPP (rIAPP-K-Bodipy FL) as monitored by FCS.

|  | *D* / µm^2^s^-1^ |
| --- | --- |
| without crowder | 138.4 ± 9.5 |
| 10 % Ficoll | 51.4 ± 0.2 |
| 20 % Ficoll | 25.2 ± 2.0 |
| 30 % Ficoll | 8,7 ± 1.3 |
| 40 % Ficoll | 2.9 ± 0.3 |
| 10 % dextran | 40.3 ± 4.0 |
| 20 % dextran | 23.8 ± 0.3 |
| 30 % dextran | 9.8 ± 1.4 |
| 40 % dextran | 3,8 ± 0.4 |
| 10 % BSA | 30.1 ± 8.2 |
| 20 % BSA | 16 6 ± 0.3 |
| 30 % BSA | 4.9 ± 1.1 |
| 40 % BSA | 2.4 ± 0.3 |
| 10 % lysozyme | 29.8 ± 2.2 |
| 20 % lysozyme | 14.5 ± 2.5 |
