# Supplementary material for: Macromolecular Crowding as a Suppressor of Human IAPP Fibril Formation and Cytotoxicity
Source: PLoS One. 2013 Jul 29;8(7):e69652. doi: 10.1371/journal.pone.0069652 (PMC3726762; doi:10.1371/journal.pone.0069652)
Supplement: Table S2 — Fraction of crowder-bound rIAPP-K-Bodipy FL as derived from the two-component 3D-diffusion analysis. (DOCX) [file pone.0069652.s007.docx]

**Table S2.** Fraction of crowder-bound rIAPP-K-Bodipy FL as derived from the two-component 3D-diffusion analysis.

|  | bound rIAPP / % |
| --- | --- |
| 10 µM Ficoll | 0.23 ± 0.03 |
| 10 µM dextran | 0.18 ± 0.04 |
| 10 µM BSA | 0.51 ± 0.05 |
| 10 µM lysozyme | 0.88 ± 0.10 |
