# Supplementary material for: Macromolecular Crowding as a Suppressor of Human IAPP Fibril Formation and Cytotoxicity
Source: PLoS One. 2013 Jul 29;8(7):e69652. doi: 10.1371/journal.pone.0069652 (PMC3726762; doi:10.1371/journal.pone.0069652)
Supplement: Table S3 — Crowding molecule and crowder concentration dependent lag times and growth rate constants for the aggregation/fibrillation process of 10 µM hIAPP. (DOCX) [file pone.0069652.s008.docx]

**Table S3.** Crowding molecule and crowder concentration dependent lag times and growth rate constants for the aggregation/fibrillation process of 10 µM hIAPP.^a^

|  | *lag time* / h | *k*_app_ / h^-1^ |
| --- | --- | --- |
| without crowder | 1.5 ± 0.1 | 2.1 ± 0.1 |
| 10 µM Ficoll | 1.4 ± 0.1 | 3.2 ± 0.3 |
| 10 % Ficoll | 1.5 ± 0.1 | 2.2 ± 0.2 |
| 20 % Ficoll | 1.4 ± 0.1 | 2.4 ± 0.1 |
| 30 % Ficoll | 1.3 ± 0.1 | 1.5 ± 0.1 |
| 10 µM dextran | 1.3 ± 0.1 | 3.2 ± 0.3 |
| 10 % dextran | 1.6 ± 0.1 | 2.3 ± 0.1 |
| 20 % dextran | 1.2 ± 0.2 | 1.8 ± 0.2 |
| 30 % dextran | -^b^ | -^b^ |
| 40 % dextran | -^b^ | -^b^ |
| 10 µM BSA | 2.9 ± 0.3 | 0.6 ± 0.0 |
| 10 % BSA | 2.5 ± 0.3 | 0.9 ± 0.1 |
| 20 % BSA | 4.7 ± 0.3 | 0.7 ± 0.1 |
| 30 % BSA | 5.1 ± 0.6 | 0.5 ± 0.1 |
| 10 µM lysozyme | 1.7 ± 0.0 | 4.2 ± 0.3 |

^a^Lag times and growth rate constants (± standard deviations) were determined as described previously [1]. ^b^Calculation of parameters is not applicable.

**References**

1. Nielsen L, Khurana R, Coats A, Frokjaer S, Brange J, et al. (2001) Effect of Environmental Factors on the Kinetics of Insulin Fibril Formation: Elucidation of the Molecular Mechanism. Biochemistry 40: 6036–6046.
