# Supplementary material for: Macromolecular Crowding as a Suppressor of Human IAPP Fibril Formation and Cytotoxicity
Source: PLoS One. 2013 Jul 29;8(7):e69652. doi: 10.1371/journal.pone.0069652 (PMC3726762; doi:10.1371/journal.pone.0069652)
Supplement: Table S4 — Heights of hIAPP oligomers and fibrils as detected by AFM. (DOCX) [file pone.0069652.s009.docx]

**Table S4.** Heights of hIAPP oligomers and fibrils as detected by AFM.^a^

|  | *fibril height* / nm | *large oligomer height* / nm | *small oligomer height* / nm |
| --- | --- | --- | --- |
| without crowder | 5.8 ±2.7 | s.n.d.^b^ | s.n.d.^b^ |
| 20 % Ficoll | 5.8 ± 2.2 | s.n.d.^b^ | s.n.d.^b^ |
| 40 % Ficoll | 5.3 ± 2.2 | s.n.d.^b^ | s.n.d.^b^ |
| 20 % dextran | 5.7 ± 2.0 | s.n.d.^b^ | s.n.d.^b^ |
| 40 % dextran | 5.5 ± 2.3 | s.n.d.^b^ | s.n.d.^b^ |
| 20 % BSA | 5.2 ± 1.0 | s.n.d.^b^ | 0.8 ± 0.4 |
| 40 % BSA | s.n.d.^b^ | 4.4 ± 0.6 | 0.9 ± 0.5 |
| 20 % lysozyme | 6.1 ± 2.0 | 4.9 ± 1.3 | 0.7 ± 0.5 |

^a^Heights are mean values ± standard deviation of *n* = 200 or *n* > 1000 fibrils or oligomeric species, respectively. ^b^Species not detected.
