# Supplementary material for: Macromolecular Crowding as a Suppressor of Human IAPP Fibril Formation and Cytotoxicity
Source: PLoS One. 2013 Jul 29;8(7):e69652. doi: 10.1371/journal.pone.0069652 (PMC3726762; doi:10.1371/journal.pone.0069652)
Supplement: Supporting Information S1 — Additional information to the ThT assay results of the samples used for atomic force microscopy (AFM) measurements. (DOCX) [file pone.0069652.s010.docx]

**Supporting Information.** Additional information to the ThT assay results of the samples used for atomic force microscopy (AFM) measurements

Higher hIAPP concentrations were needed for the AFM measurements compared to the ThT fluorescence spectroscopic assay. To this end, aliquots of 50 µM hIAPP were taken after 15 h of incubation in crowded solutions under ThT assay conditions and used for the measurements. The corresponding time-laps ThT data are displayed in Figure S1 showing similar results as detected for the lower 10 µM hIAPP concentration (manuscript Fig. 4) with minor changes in the kinetics and the strength of the inhibitory effect of the crowder, only. In line with the results for 10 µM hIAPP, no changes in the kinetics compared to the hIAPP aggregation in the absence of crowding reagents were found for 50 µM hIAPP in 20% Ficoll and dextran solutions, whereas extended elongation phases were detected for 40% Ficoll and dextran compared to the total inhibition of fibrillation at the lower 10 µM hIAPP concentration. Prolonged lag as well as elongation phases were obtained for 50 µM hIAPP in 20% BSA solution, and 40% of BSA totally inhibited the hIAPP aggregation, which is again in line with the results for 10 µM hIAPP. Total inhibition of hIAPP fibrillation as by lysozyme at *c*(hIAPP) = 10 µM was not detected for a concentration of 50 µM hIAPP, however a strong reduction of the ThT intensity, indicating much less fibril formation, was identified for the higher hIAPP concentration as well. This explains the findings of very few fibrils in the AFM image of 50 µM hIAPP in 20% lysozyme.
